# Supplementary material for: Additive manufacturing of 3D nano-architected metals
Source: Nat Commun. 2018 Feb 9;9:593. doi: 10.1038/s41467-018-03071-9 (PMC5807385; doi:10.1038/s41467-018-03071-9)
Supplement: Supplementary file 1 — Supplementary Information [file 41467_2018_3071_MOESM1_ESM.pdf]

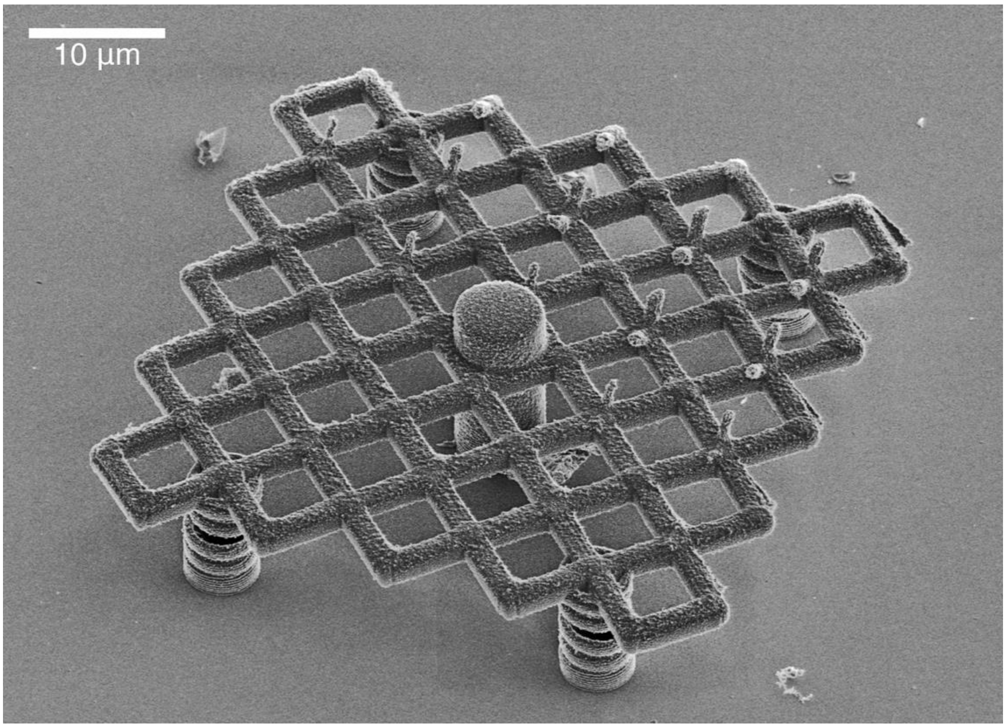

Supplementary Figure 1

SEM image of a representative supporting structure used to decouple the part from the substrate during pyrolysis.

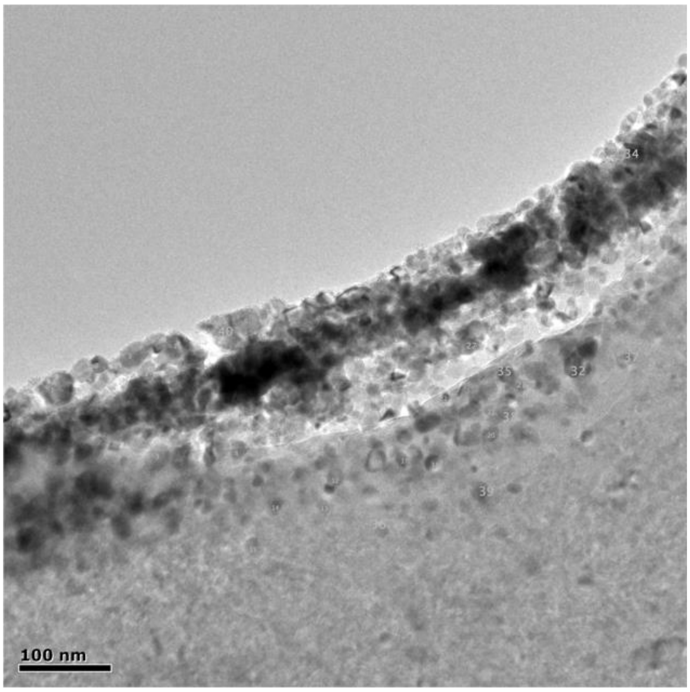

Supplementary Figure 2

Bright-field TEM image of a printed nickel beam overhanging a hole in the silicon nitride membrane. Numbers correspond to the grains used for mean particle size calculation.

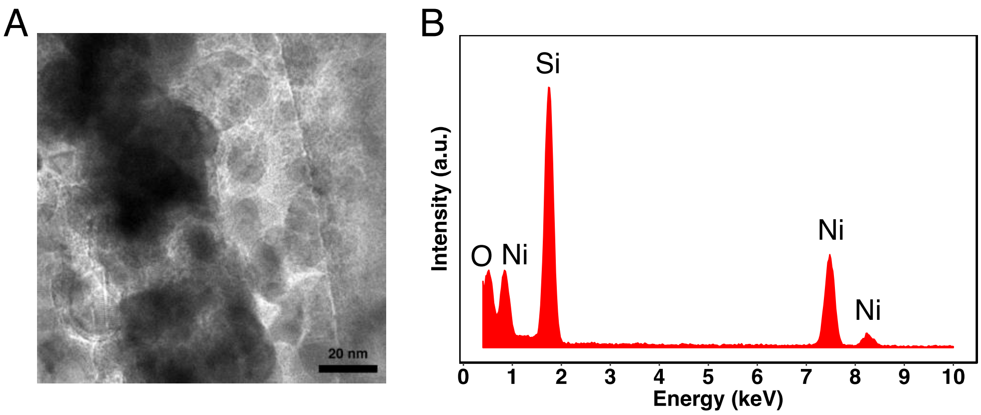

Supplementary Figure 3

TEM EDS characterization of the printed beams. (**A**) The region of TEM where the EDS spectrum was taken from. (**B**) EDS spectrum shows high nickel content.

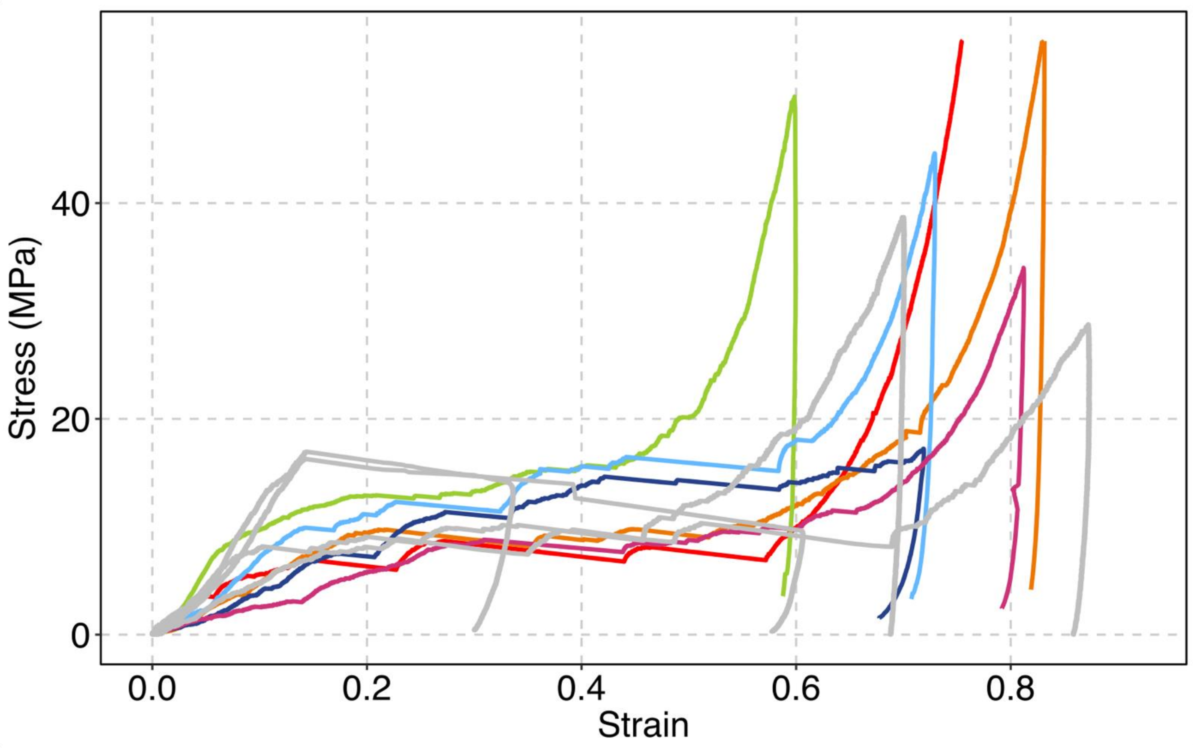

Supplementary Figure 4. Stress vs. strain of additional six nanolattices. The original data shown in Fig. 4 in the main manuscript is presented in gray, additional data is shown in color.

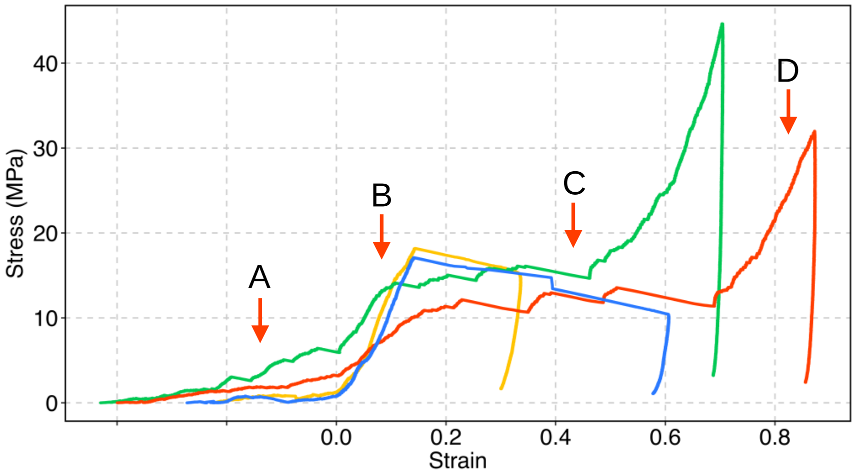

Supplementary Figure 5

Stress-strain diagram showing full compression data including the toe region for four representative nickel nanolattices. Letters on the graph correspond to (A) the toe region, (B) the elastic region, (C) layer-by-layer collapse, and (D) densification.

Supplementary Table 1

Particle sizes collected from the bright-field TEM image shown in Supplementary Fig. 2

N	Size, nm	N	Size, nm	N	Size, nm	N	Size, nm	N	Size, nm
1	37.24	9	33.05	17	17.43	25	12.14	33	12.82
2	19.47	10	19.86	18	18.98	26	16.59	34	29.34
3	33.79	11	16.73	19	23.25	27	25.43	35	21.67
4	25.29	12	19.59	20	22.16	28	16.6	36	14.88
5	30.55	13	19.03	21	15.15	29	16.61	37	18.95
6	17.24	14	20.41	22	19.26	30	17.98	38	19.95
7	31.06	15	22.85	23	16.61	31	14.29	39	21.81
8	19.04	16	26.94	24	12.52	32	27.28	40	30.72

Supplementary Table 2

Specific strength of solid-beam metal lattices fabricated using metal AM processes and electroplating into a template

Material	Lattice Type	Process	Beam diameter, µm	Strength, MPa	Relative density	Material density, g/cm^3	Lattice density, g/cm^3	Specific strength, $MPa/(g/cm^3)$	Ref.
Ti-6Al-4V	Cubic	Electron Beam Melting (EBM)	810	23.70	0.063	4.43	0.26	84.92	1
			970	34.70	0.078		0.32	100.42	
			1480	89.10	0.159		0.65	126.50	
			1780	180.20	0.216		0.88	188.32	
Ti-6Al-4V	Topology-optimized	Selective Laser Melting (SLM)	406	30.00		n/a	0.50	60.00	2
AlSi10Mg	Diamond	Direct Metal Laser Sintering (DMLS)	405	1.42	0.050	2.67	0.12	10.63	3
			502	4.72	0.075		0.17	23.54	
			659	8.54	0.100		0.23	31.98	
			765	12.61	0.125		0.29	37.76	
			862	17.40	0.150		0.35	43.45	
Stainless steel 316L	BCC	Selective Laser Melting (SLM)	162	0.20	0.023	n/a	0.19	1.05	4
			181	0.33	0.029		0.23	1.43	
			181	0.33	0.029		0.23	1.43	
			197	0.45	0.034		0.28	1.61	
			197	0.45	0.035		0.28	1.61	
			212	0.58	0.040		0.32	1.81	
			212	0.60	0.041		0.33	1.82	
			186	0.38	0.031		0.25	1.52	
			210	0.55	0.039		0.31	1.77	
			230	0.79	0.047		0.38	2.08	
			249	1.00	0.055		0.44	2.27	
			165	0.32	0.030		0.24	1.33	
			166	0.33	0.032		0.26	1.27	
			186	0.47	0.036		0.29	1.62	
			188	0.46	0.034		0.28	1.64	
			222	0.83	0.047		0.38	2.18	
			211	0.73	0.043		0.34	2.15	
Silver	Octahedral	Pointwise Spatial Printing	35	0.60	0.065	n/a	0.50	1.20	5
			38	1.27	0.270		1.74	0.73	
NiTi	Octahedral	Selective laser melting (SLM)	248	21.00	0.252	6.45	1.63	12.92	6
	Cellular gyroid		298	29.00	0.252		1.63	17.84	
	Sheet gyroid		210	44.00	0.266		1.72	25.65	
Copper	Octet	Electroplating into a template	1.011	221.84	0.53	8.96	4.72	46.95	7
			1.025	136.29	0.54		4.82	28.28	
			1.050	158.23	0.56		5.02	31.52	
			1.098	179.54	0.60		5.35	33.54	
			1.164	154.11	0.65		5.80	26.57	
			1.178	241.14	0.66		5.91	40.82	
			1.218	296.10	0.69		6.18	47.93	

			1.323	266.56	0.76		6.83	39.02
			1.340	271.62	0.77		6.93	39.18
			1.378	332.70	0.80		7.16	46.48
Nickel	Octet	This work	0.30	18.17		8.91	2.52	7.20
			0.30	17.08			2.55	6.71
			0.28	8.91			2.60	3.42
			0.27	8.18			2.75	2.98
			0.35	6.94			3.03	2.29
			0.42	9.71			3.30	2.95
			0.32	12.31			2.94	4.19
			0.36	12.87			4.01	3.21
			0.33	7.50			3.65	2.05
			0.32	8.81			3.03	2.91

Supplementary Table 3

Comparison of minimum feature sizes for metal additive manufacturing technologies

#	Technology/reference	Machine/company/setup	Layer thickness range, µm		Lateral feature range, µm		Beam diameter, µm	Ref.
			Min	Max	Min	Max		
1	Selective Laser Melting (SLM)	SLM Solutions SLM 125	20	75	140			8
2	Direct Metal Laser Sintering (DMLS)	EOS	20	80	300	700		9
3	Electron Beam Melting (EBM)	Arcam	50	200	500			9
4	LaserCUSING	CONCEPT Laser M1 Cusing	20	80				9
5	Digital Part Materialization	ExOne M-Flex	100		60	63.1		9,10
6	Direct Metal Deposition (DMD)	POM DMD 105D	100	1600				9
7	Laser Engineered Net Shaping (LENS)	Optomec LENS MR-7	25				250	9
8	Laser Metal Deposition (LMD)	BeAM MOBILE Machine	100		800	1200		11
9	Rapid Plasma Deposition	NORSK TITANIUM MERKE IV	3000	4000	8000	12000		12
10	Electron Beam Free Form Fabrication (EBF3)	Sciaky EBAM 300			3000	10000		13,14
11	Ultrasonic Consolidation (UC)	Fabrisonic SonicLayer 7200	1500	2500				15
12	Metal Powder Bed Fusion	Renishaw AM250	20	100				16
13	Direct Laser Forming	Trumpf TrumaForm LF130	50				200	17
14	Digital Metal	Höganäs	20		40			18,19
15	Electrochemical Fabrication (EFAB)	Microfabrica	4		10			20
16	Regenfuss et al., 2007	Powder-based	10		55	60		21
17	Kullman et al., 2012	Wire-based			50	250		22
18	Saleh et al., 2017	Inkjet-based	10	20	30	55		5
19	Takai et al., 2014	Local electrophoresis			0.5	2.0		23
20	Hirt et al., 2016	Local electroplating	0.25		0.8	5.0		24
21	Visser et al., 2015	Laser-Induced Forward Transfer (LIFT)			4	6		25
22	Scylar-Scott et al., 2016	Laser-assisted Direct Ink Writing (DIW)			0.6	20		26
23	This work	Nanoscribe Photonic Professional GT		0.03	0.025	0.4		

Supplementary Table 4

Comparison of linear and volumetric throughputs of representative micro-scale metal additive manufacturing technologies (data adopted from ref.[27])

#	Technology	Material	Feature size, µm	Writing speed*	Ref.
1	Direct Ink Writing (DIW)	Ag	0.6-20	500-2000 µm s^{-1}	26
2	Electrohydrodynamic (EHD) Printing	Ag, Co, Cu	0.7-3.0	0.16-3.3 µm s^{-1}	28
3	Laser-Induced Forward Transfer (LIFT)	Au, Cu	4.0-6.0	3000 μm^3 s^{-1}	25
4	Focused Electron Beam Induced Deposition (FEBID)	Pt	0.15-0.23	0.0002-0.0009 μm^3 s^{-1}	29
5	Cryo-FEBID	Pt	0.022-0.31	10 μm^3 s^{-1}	30
6	Meniscus-confined electroplating	Cu	12.0-15.0	0.18-0.4 µm s^{-1}	31
7	Local electrophoretic deposition	Au	0.5-2.0	0.30-0.67 µm s^{-1}	23
8	This work	Ni	0.025-0.4	4000-6000 µm s^{-1}	

*Volumetric (μm^3 s^{-1}) or linear (µm s^{-1}) writing speed is given when available

Supplementary References

1. Hernández-Nava, E. *et al.* The effect of defects on the mechanical response of Ti-6Al-4V cubic lattice structures fabricated by electron beam melting. *Acta Mater.* **108,** 279–292 (2016).
2. Challis, V. J. *et al.* High specific strength and stiffness structures produced using selective laser melting. *Mater. Des.* **63,** 783–788 (2014).
3. Yan, C. *et al.* Evaluation of light-weight AlSi10Mg periodic cellular lattice structures fabricated via direct metal laser sintering. *J. Mater. Process. Technol.* **214,** 856–864 (2014).
4. Tsopanos, S. *et al.* The Influence of Processing Parameters on the Mechanical Properties of Selectively Laser Melted Stainless Steel Microlattice Structures. *J. Manuf. Sci. Eng.* **132,** 41011–41012 (2010).
5. Saleh, M. S., Hu, C. & Panat, R. Three-dimensional microarchitected materials and devices using nanoparticle assembly by pointwise spatial printing. *Sci. Adv.* **3,** (2017).
6. Speirs, M., Van Hooreweder, B., Van Humbeeck, J. & Kruth, J.-P. Fatigue behaviour of NiTi shape memory alloy scaffolds produced by SLM, a unit cell design comparison. *J. Mech. Behav. Biomed. Mater.* **70,** 53–59 (2017).
7. Wendy Gu, X. & Greer, J. R. Ultra-strong architected Cu meso-lattices. *Extrem. Mech. Lett.* **2,** 7–14 (2015).
8. Selective Laser Melting Machine. Available at: https://slm-solutions.com/sites/default/files/attachment/page/2016/11/w_slm125_en.pdf. (Accessed: 13th April 2017)
9. Karunakaran, K. P., Bernard, A., Suryakumar, S., Dembinski, L. & Taillandier, G. Rapid manufacturing of metallic objects. *Rapid Prototyp. J.* **18,** 264–280 (2012).
10. M-Flex. Available at: http://www.exone.com/Portals/0/Systems/M-Flex/X1_MFlex_US.pdf. (Accessed: 13th April 2017)
11. BeAM Mobile Machine. Available at: http://www.beam-machines.fr/uploads/files/leaflets mobile en_10.02.2016.pdf. (Accessed: 13th April 2017)
12. NORSK TITANIUM MERKE IV. Available at: http://www.norsktitanium.com/wp-content/uploads/2016/07/Norsk_Titanium_Brochure.pdf. (Accessed: 13th April 2017)
13. Metal Additive Manufacturing Systems - EBAM® Systems | Sciaky. Available at: http://www.sciaky.com/additive-manufacturing/metal-additive-manufacturing-systems. (Accessed: 13th April 2017)
14. Sciaky Discusses 2015 3D Printing Plans - 3D Printing Industry. Available at: https://3dprintingindustry.com/news/sciakys-john-ohara-ebam-technologys-3d-printing-projects-2015-40284/. (Accessed: 13th April 2017)
15. Fabrisonic’s SonicLayer 7200 Fully Automated System is a large-format metal 3D printer. Available at: http://www.plantservices.com/vendors/products/2015/fabrisonics-soniclayer-7200-fully-automated-system/. (Accessed: 13th April 2017)
16. Kair, A. B. & Sofos, K. *Additive Manufacturing and Production of Metallic Parts in Automotive Industry A Case Study on Technical , Economic and Environmental Sustainability Aspects*. (2014).
17. Brandl, E., Heckenberger, U., Holzinger, V. & Buchbinder, D. Additive manufactured AlSi10Mg samples using Selective Laser Melting (SLM): Microstructure, high cycle fatigue, and fracture behavior. *Mater. Des.* **34,** 159–169 (2012).
18. Digital Metal. Additive manufacturing of small and complex metal parts. Available at: https://www.hoganas.com/globalassets/media/sharepoint-documents/BrochuresanddatasheetsAllDocuments/Additivemanufacturingofsmallandc

omplexmetalparts_July_2016_1666HOG.pdf. (Accessed: 13th April 2017)
19. Vaezi, M., Seitz, H. & Yang, S. A review on 3D micro-additive manufacturing technologies. *Int. J. Adv. Manuf. Technol.* **67,** 1721–1754 (2013).
20. Cohen, A., Chen, R., Frodis, U., Wu, M. & Folk, C. Microscale metal additive manufacturing of multi-component medical devices. *Rapid Prototyp. J.* **16,** 209–215 (2010).
21. Regenfuss, P. *et al.* Principles of laser micro sintering. *Rapid Prototyp. J.* **13,** 204–212 (2007).
22. Kullmann, C. *et al.* 3D micro-structures by piezoelectric inkjet printing of gold nanofluids. *J. Micromechanics Microengineering* **22,** 55022 (2012).
23. Takai, T., Nakao, H. & Iwata, F. Three-dimensional microfabrication using local electrophoresis deposition and a laser trapping technique. *Opt. Express* **22,** 28109–28117 (2014).
24. Hirt, L. *et al.* Template-Free 3D Microprinting of Metals Using a Force-Controlled Nanopipette for Layer-by-Layer Electrodeposition. *Adv. Mater.* **28,** 2311–2315 (2016).
25. Visser, C. W. *et al.* Toward 3D Printing of Pure Metals by Laser-Induced Forward Transfer. *Adv. Mater.* **27,** 4087–4092 (2015).
26. Skylar-Scott, M. A., Gunasekaran, S. & Lewis, J. A. Laser-assisted direct ink writing of planar and 3D metal architectures. *Proc. Natl. Acad. Sci.* **113,** 6137–6142 (2016).
27. Hirt, L., Reiser, A., Spolenak, R. & Zambelli, T. Additive Manufacturing of Metal Structures at the Micrometer Scale. *Adv. Mater.* 1604211–n/a (2017). doi:10.1002/adma.201604211
28. An, B. W. *et al.* High-Resolution Printing of 3D Structures Using an Electrohydrodynamic Inkjet with Multiple Functional Inks. *Adv. Mater.* **27,** 4322–4328 (2015).
29. Plank, H., Gspan, C., Dienstleder, M., Kothleitner, G. & Hofer, F. The influence of beam defocus on volume growth rates for electron beam induced platinum deposition. *Nanotechnology* **19,** 485302 (2008).
30. Bresin, M., Toth, M. & Dunn, K. A. Direct-write 3D nanolithography at cryogenic temperatures. *Nanotechnology* **24,** 35301 (2013).
31. Seol, S. K. *et al.* Electrodeposition-based 3D Printing of Metallic Microarchitectures with Controlled Internal Structures. *Small* **11,** 3896–3902 (2015).
