# Supplementary material for: Additive manufacturing of 3D nano-architected metals
Source: Nat Commun. 2018 Feb 9;9:593. doi: 10.1038/s41467-018-03071-9 (PMC5807385; doi:10.1038/s41467-018-03071-9)
Supplement: Supplementary file 3 — Description of Additional Supplementary Files [file 41467_2018_3071_MOESM3_ESM.docx]

**Description of Additional Supplementary Files**

File Name: Supplementary Movie 1

Description: *In-situ* video (played at 40x speed) of uniaxial compression of a nickel octet nanolattice with ~2 μm unit cells and 300-400 nm-diameter beams to ~85% strain. Elastic deformation (up to ~15% strain) is followed by layer-by-layer collapse (up to ~70% strain) and densification. The nanolattice does not recover after compression.
